# An iodide-containing covalent organic framework for enhanced radiotherapy†

**DOI:** 10.1039/d3sc00251a

**Published:** 2023-03-06

**Authors:** Le-Le Zhou, Qun Guan, Wei Zhou, Jing-Lan Kan, Yu-Bin Dong

**Affiliations:** a College of Chemistry, Chemical Engineering and Materials Science, Collaborative Innovation Center of Functionalized Probes for Chemical Imaging in Universities of Shandong, Key Laboratory of Molecular and Nano Probes, Ministry of Education, Shandong Normal University Jinan 250014 China yubindong@sdnu.edu.cn; b Department of Oncology, Shandong Provincial Hospital Affiliated to Shandong First Medical University Jinan 250021 China

## Abstract

Metal-free radiosensitizers, particularly iodine, have shown promise in enhancing radiotherapy due to their suitable X-ray absorption capacities and negligible biotoxicities. However, conventional iodine compounds have very short circulating half-lives and are not retained in tumors very well, which significantly limits their applications. Covalent organic frameworks (COFs) are highly biocompatible crystalline organic porous materials that are flourishing in nanomedicine but have not been developed for radiosensitization applications. Herein, we report the room-temperature synthesis of an iodide-containing cationic COF by the three-component one-pot reaction. The obtained TDI-COF can be a tumor radiosensitizer for enhanced radiotherapy by radiation-induced DNA double-strand breakage and lipid peroxidation and inhibits colorectal tumor growth by inducing ferroptosis. Our results highlight the excellent potential of metal-free COFs as radiotherapy sensitizers.

## Abstract 2

An iodide-containing cationic covalent organic framework as a nonmetallic radiosensitizer increases X-ray deposition and enhances radiotherapy of colon cancer *in vitro* and *in vivo* by inducing ferroptosis.

## Graphical abstract

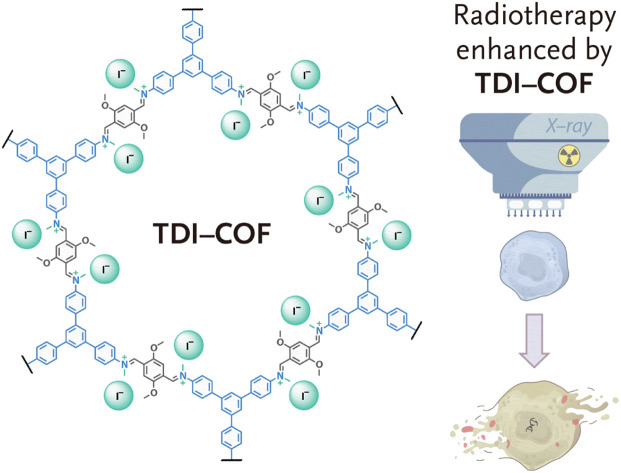

## Introduction

More than half of the cancer patients are subjected to radiotherapy at some timepoint during disease progression; this technique uses high-energy X-rays to damage cancer cells, and is one of the most cost-effective cancer treatment options.^1^ However, the dose and efficacy of radiotherapy are limited by normal tissue toxicity.^2^ Radiosensitizers concentrate radiation energy within cancerous tissue or destroy tumor resistance to X-rays, which can improve the efficacy of radiotherapy without increasing the radiation dose, and potentially enhance the radiotherapeutic window, particularly for malignancies with high risks of regional recurrence, such as gastrointestinal cancers.^3^ Despite extensive (pre)clinical data that demonstrate the radiosensitizing properties of chemotherapeutic drugs, the intolerable side effects of chemotherapy are clinically limiting its applications.^4–6^

Nanoparticles containing high-*Z* elements, such as Au nanoparticles,^7–11^ Bi(iii) chalcogenides,^12–15^ and metal–organic frameworks (MOFs),^16–20^ have recently been used as radiosensitizers. While these inorganic nanomaterials provide large X-ray absorption cross-sections that amplify the radiation energy deposited in tumor tissue and improve radiobiological effects, their biosafety has been questioned.^21^ Hence, there is an urgent need to develop metal-free radiosensitizers that are highly biocompatible; in this regard, iodine compounds are noteworthy candidates.^22–24^ For example, iopromide and iohexol have high X-ray absorption capacities but are suitable for computed tomography (CT) imaging rather than radiosensitization due to poor vessel-wall penetration, extremely short circulatory half-lives, and low tumor retention.

Covalent organic frameworks (COFs), which are crystalline porous polymeric materials with well-defined chemical structures,^25–30^ have attracted extensive research interest in the tumor nanomedicine field, and have been used in drug delivery,^31–36^ phototherapy,^37–46^ and immunotherapy^47–52^ because they are versatile and biocompatible. Recently, AgI@COF-TpBpy was used as a delivery vehicle for radioiodine in brachytherapy, showing a long tumor retention time and effective cancer cell killing performance;^53^ however, to our knowledge, the potential of a metal-free COF itself as a radiosensitizer in radiotherapy has not been exploited.^54–56^

**Fig. 1:**
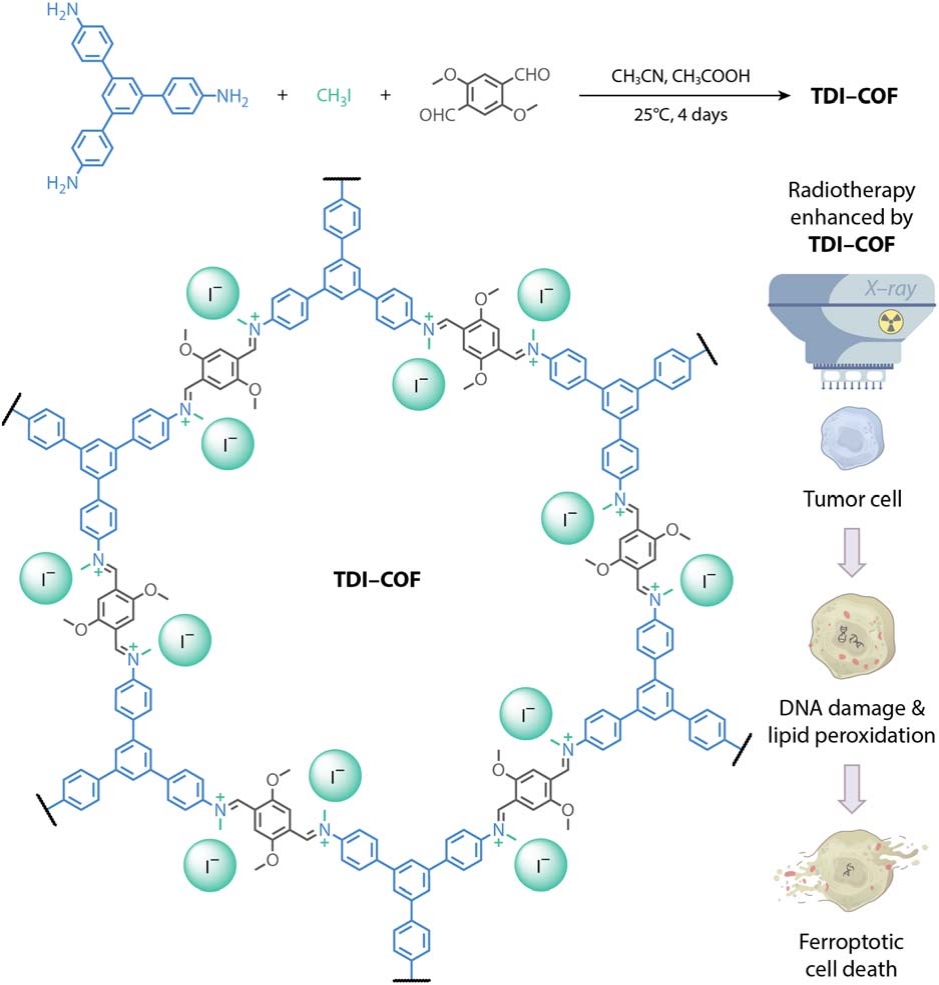
Synthesis of an iodide-containing and iminium-linked TDI-COF as a radiosensitizer for improving radiotherapy efficacy *via* iodide-promoted X-ray deposition.

In this contribution, we reported a cationic COF with iodide counterions prepared by a three-component *in situ* reaction.^57^ The generated TDI-COF can be a tumor radiosensitizer to enhance colorectal cancer radiotherapy (Fig. 1). The good X-ray absorption capacity of TDI-COF allowed it to promote radiation-induced DNA damage and lipid peroxidation, and induce ferroptosis that inhibits cell proliferation and tumor growth. To our knowledge, this study is not only the first example of metal-free COFs for radiotherapy, but also highlights their great potential as radiotherapy radiosensitizers.

## Results and discussion

**Fig. 2:**
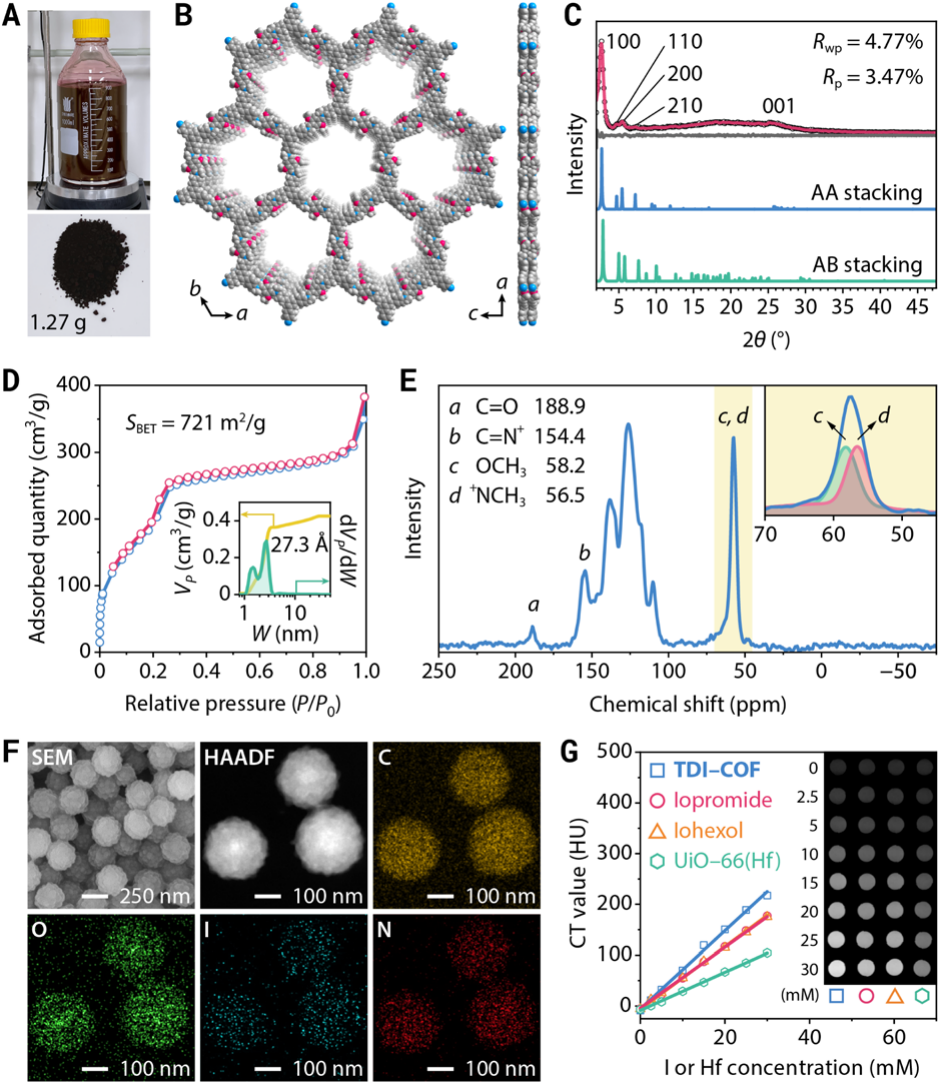
Synthesis and characterization of TDI-COF. (A) Gram-scale synthesis of TDI-COF. (B) Top and side views of the crystal structure of TDI-COF. (C) Experimental (black dots), Pawley-refined (red), and simulated (blue and green) PXRD patterns and difference plot (gray). (D) N_2_ adsorption–desorption isotherm. Inset: cumulative pore volume profile (yellow) and pore size distribution (green). (E) ^13^C CP-MAS NMR spectrum and deconvolution. (F) SEM and HAADF-STEM images and elemental maps. (G) CT images and the corresponding attenuation plots.

TDI-COF, which is linked through iminium moieties,^58^ was prepared by the one-pot *in situ* reaction of 1,3,5-tris(4-aminophenyl)benzene (TPB), iodomethane, and 2,5-dimethoxyterephthalaldehyde (DMTP) in acetonitrile with acetic acid at 25 °C for 4 days (Fig. 2A). The formation of a model compound of *N*-benzylidene-*N*-methylbenzenaminium iodide in acetonitrile solution implied the rationality of the polymerization reaction (Fig. S1A, ESI†). Inductively coupled plasma–mass spectrometry (ICP-MS) and elemental analysis revealed that TDI-COF contains 36.7 ± 1.3 wt% iodine, which is consistent with the C_78_H_60_N_6_O_6_(CH_3_I)_5.8_ composition and is very close to the theoretical C_78_H_60_N_6_O_6_(CH_3_I)_6_ formula (Fig. S1B, ESI†).

The crystal structure of TDI-COF was determined by powder X-ray diffractometry (PXRD) in combination with computational simulations using the Forcite module in *BIOVIA Materials Studio 2018* to build an initial model,^59–61^ followed by density functional based tight binding (DFTB+) calculations to optimize the conformation of the 2D layer and the stacking mode (Fig. 2B).^62–64^ The structure of TDI-COF was built with the **hcb** topology in space group *P*3 (No. 143) and was further refined from the PXRD pattern by Pawley refinement (Fig. 2C). The layers are stacked in an eclipsed AA mode (Table S1, ESI†), with cell parameters *a* = *b* = 37.45 Å, *c* = 3.46 Å, *α* = *β* = 90°, and *γ* = 120°, and good agreement factors (*R*_p_ = 3.47% and *R*_wp_ = 4.77%). In contrast, the simulated diffraction pattern of the staggered AB-stacking model deviates from the experimental data for the (100), (110), (200), and (001) peaks, eliminating the AB-stacking structure (Table S2, ESI†).

Furthermore, its permanent porosity was determined by the N_2_ adsorption–desorption isotherm acquired at 77 K; the distinct step observed at *P*/*P*_0_ = 0.18–0.26 is the result of mesopore filling and is consistent with a type IV isotherm (Fig. 2D). The Brunauer–Emmett–Teller (BET) surface area was determined to be *S*_BET_ = 721 m^2^ g^−1^ with a total pore volume of 0.42 cm^3^ g^−1^. The pore size distribution of TDI-COF calculated by nonlocal density functional theory revealed cylindrical pores that are 2.73 nm in diameter,^65^ in good agreement with its simulated crystal structure, and micropores with a diameter of 1.48 nm that may be associated with local bonding defects and disorder stacking structures.^66,67^ Notably, the cumulative pore volume profile of TDI-COF indicated that the contribution of the 2.73 nm mesopore is greater than that of the 1.48 nm micropore, indicating the predominance of the mesopore and the satisfactory structural integrity of TDI-COF (Fig. 2D).

The chemical structure of TDI-COF was clearly identified by comparison with the unmethylated imine-linked TPB-DMTP-COF produced from TPB and DMTP (Fig. S1C and D, ESI†).^68^ The iminium C of TDI-COF was observed at 154.4 ppm in its ^13^C cross-polarization-magic angle spinning nuclear magnetic resonance (CP-MAS NMR) spectrum, fully confirming the formation of an iminium linkage (Fig. 2E). The nonexistent imine C (149.4 ppm) and very weak aldehyde C (188.9 ppm) characteristic peaks are again indicative of a high-yield iminium formation (Fig. S1E, ESI†). The methyl C signal was deconvoluted into two peaks with nearly identical intensities that respectively correspond to N^+^CH_3_ (56.5 ppm) and OCH_3_ (58.2 ppm). The formation of the iminium linkage was further supported by Fourier-transform infrared spectroscopy, with a characteristic C=N^+^ stretching vibration at 1645 cm^−1^ and negligible peaks of the residual CHO and C=N at 1682 and 1617 cm^−1^, respectively (Fig. S1F, ESI†).^69^ Furthermore, the high-resolution N 1s X-ray photoelectron spectroscopy (XPS) profile showed a symmetrical peak at 401.2 eV that further evidenced the formation of a C=N^+^ moiety (Fig. S1G, ESI†). In addition, two XPS peaks associated with I^−^ are located at 619.3 and 630.8 eV, with a well-separated 11.5 eV spin–orbit component, and no impurity peaks associated with IO_3_^−^, C–I bonds, or polyiodide species (*e.g.*, I_3_^−^ and I_5_^−^) were detected, confirming that the only form of iodine present is I^−^ (Fig. S1H, ESI†).^70,71^ Consistent with this, the characteristic stretching vibration bands of the polyiodide species were also not detected in the Raman spectrum of TDI-COF (Fig. S1I, ESI†).^72^ Thermogravimetric analysis showed that TDI-COF is thermally stable up to approximately 280 °C, demonstrating the absence of I_2_ species in the pores (Fig. S1J, ESI†).

Transmission electron microscopy (TEM) and scanning electron microscopy (SEM) images revealed that TDI-COF possesses a uniform nanospherical morphology with a particle size of 243 ± 13 nm (Fig. S1K and L, ESI†). High-resolution TEM images revealed rough nanoparticles composed of small flakes, with well-defined lattice fringes (Fig. S1M, ESI†). The fast Fourier transform of the selected area showed a twofold symmetric pattern with the expected repeat distance of 0.34 nm, which is consistent with the π–π stacking distance calculated from the simulated AA-stacking structure (Fig. S1N, ESI†). In addition, selected area electron diffraction patterns showed a diffraction ring that was indexed to the (001) plane, revealing the acceptable crystallinity and the polycrystalline nature of TDI-COF (Fig. S1O, ESI†). High-angle annular dark-field scanning transmission electron microscopy (HAADF-STEM) and element maps indicated that C, O, I, and N are uniformly distributed in the nanoparticles (Fig. 2F). Moreover, time-dependent dynamic light scattering, zeta potential analysis, and PXRD measurements showed that TDI-COF has good dispersibility and chemical stability in phosphate-buffered saline (PBS), RPMI-1640 medium, and fetal bovine serum (Fig. S2, ESI†).

The high iodide content endows TDI-COF with an effective X-ray absorption capacity that was assessed by CT, which revealed a good linear relationship between the CT value and the TDI-COF iodide concentration in PBS. The obtained specific CT value of 7.69 ± 0.19 HU mM^−1^ (I equiv.) is clearly higher than those of iopromide (5.97 ± 0.06 HU mM^−1^) and iohexol (6.15 ± 0.04 HU mM^−1^), which are clinical CT contrast media, and significantly higher than that of UiO-66(Hf), a Hf-based MOF with a CT value of 3.75 ± 0.05 HU mM^−1^ (Hf equiv.) (Fig. 2G).^73–75^

**Fig. 3:**
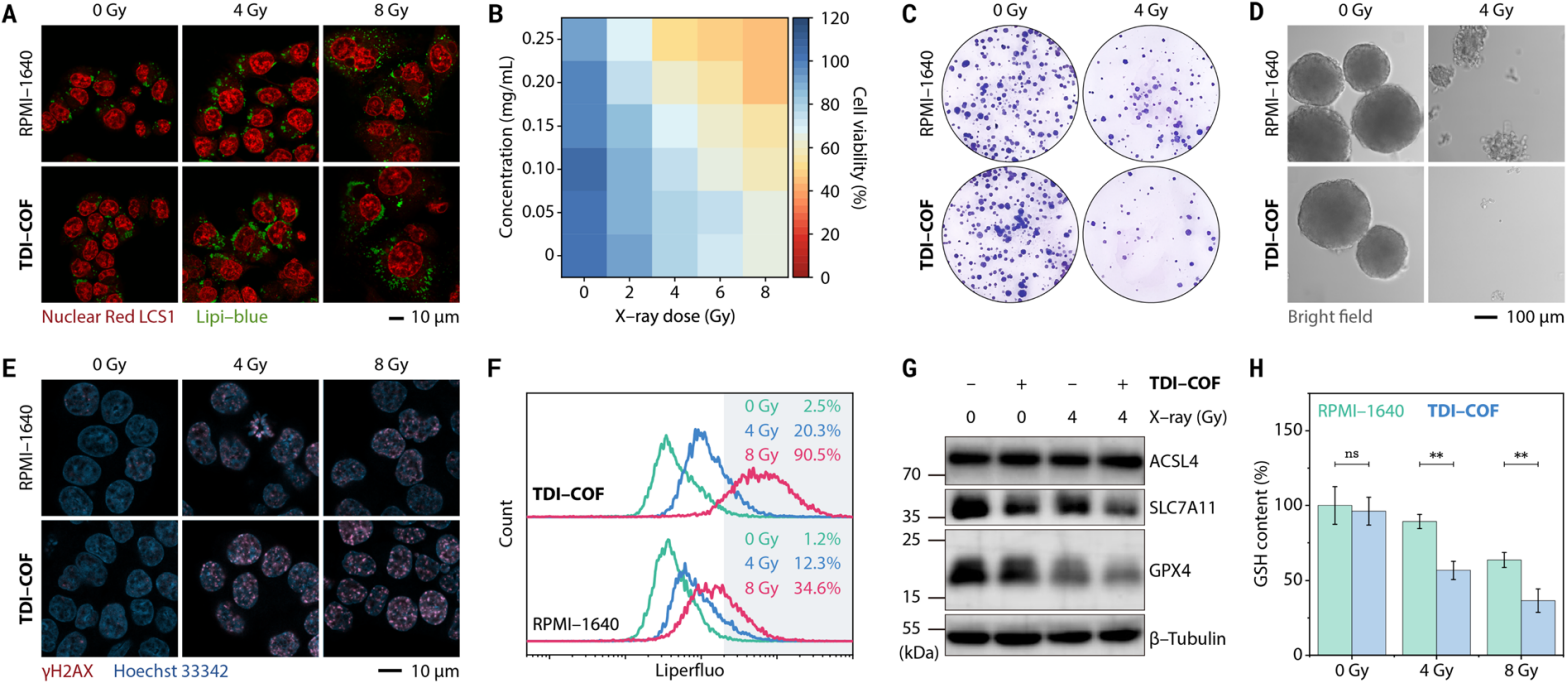
Ferroptosis-related radiotherapy of HCT-116 cells pretreated with TDI-COF (0–0.25 mg mL^−1^) for 4 h and exposed to X-ray radiation (0–8 Gy). (A) Confocal laser scanning microscopy images showing cellular morphological changes and increased lipid droplet numbers. (B) CCK-8 cell viability assay. (C) Clonogenic assay. (D) Multicellular tumor spheroid formation assay. (E) Confocal laser scanning microscopy images following γH2AX immunofluorescence staining. (F) Flow cytometric analysis of intracellular lipid peroxides. (G) Western blots of ferroptosis-related proteins. (H) Intracellular GSH levels. Data are presented as means ± SDs (*n* = 3) and compared by two-way analysis of variance (ANOVA) followed by Bonferroni's multiple comparison test. **p < 0.01; ns, no significance (*p* > 0.05).

Due to its nanoscale size and electropositive framework, TDI-COF enters tumor cells through energy-dependent pinocytosis (Fig. S3A, ESI†).^76^ Iodide ions attached to the TDI-COF crystalline framework exhibit better membrane permeability than iodinated CT contrast agents and free iodide ions (Fig. S3B, ESI†). This is because the cellular uptake of TDI-COF is independent of ion transporters^77^ and to some extent bypasses the homeostatic regulation of anions. Notably, TDI-COF was observed to increase X-ray deposition in tumor cells, thereby enhancing cellular damage. Specifically, HCT-116 colorectal cancer cells treated with TDI-COF and subsequently exposed to X-rays exhibited significantly altered morphologies (Fig. 3A), including swelling, enlarged and vacuolated nuclei, and increased lipid droplet numbers, consistent with previous reports.^78–80^ Cell viability assays further revealed that cell damage depended on the TDI-COF concentration and X-ray dose (Fig. 3B). In clonogenic assays, TDI-COF consistently showed improved radiotherapy performance, suppressing both the number and size of the formed cell colonies more significantly than X-ray exposure alone (Fig. 3C). Moreover, TDI-COF pretreatment and subsequent 4 Gy X-ray radiation almost completely inhibited the ability of HCT-116 cells to form multicellular spheres under 3D-culture conditions, demonstrating the potentiating effect of TDI-COF as a radiosensitizer toward X-ray-induced cell damage (Fig. 3D). Notably, TDI-COF affected cells negligibly in the absence of X-rays, highlighting its high biosafety.

The cell death mechanism induced by TDI-COF-enhanced radiotherapy was next explored. As a highly sensitive and specific biomarker for early-stage DNA double-strand breakage,^81^ γH2AX, the Ser139-phosphorylated product of H2A histone family member X, is more highly expressed in HCT-116 cells co-treated with TDI-COF and X-rays than in HCT-116 cells treated with X-rays alone, from which we reasoned that TDI-COF could increase DNA damage caused by radiotherapy (Fig. 3E). Liperfluo and 2′,7′-dichlorodihydrofluorescein diacetate (DCFH-DA) staining revealed that the TDI-COF treatment respectively increased the levels of intracellular lipid peroxides and reactive oxygen species (ROS) upon X-ray radiation, especially under high-dose conditions, indicative of redox dyshomeostasis (Fig. 3F and S4A, ESI†).^82,83^ In addition, we also examined ferroptosis-related protein expression given the close relationship between lipid peroxidation and ferroptosis (Fig. 3G).^84,85^ As a result, radiotherapy led to a decreased glutathione peroxidase 4 (GPX4) level, consistent with the central regulatory defense mechanism against cell ferroptosis,^86,87^ which was further exacerbated by TDI-COF. Interestingly, co-treatment with TDI-COF and X-rays also slightly decreased the expression of solute carrier family 7 member 11 (SLC7A11), which regulated ferroptosis by translocating extracellular cystine into cells.^88^ GPX4 and SLC7A11 downregulation are both contributing factors for ferroptosis. Radiotherapy negligibly affected the level of acyl-coenzyme A synthetase long-chain family member 4 (ACSL4).^89^ The intracellular level of glutathione (GSH), a major antioxidant, decreased consistently with increasing X-ray dose and was further downregulated by TDI-COF treatment (Fig. 3H). Levels of malondialdehyde, a lipid-peroxide breakdown product and a biochemical marker of ferroptosis,^90^ were significantly higher after radiotherapy (Fig. S4B, ESI†). As expected, TDI-COF treatment alone did not alter GSH and malondialdehyde levels. Furthermore, TDI-COF-enhanced radiotherapy failed to activate caspase 3 under the same conditions, suggesting that an apoptotic mechanism is not involved in cell death (Fig. S4C, ESI†). The ferroptosis mechanism was further evidenced in cell rescue experiments (Fig. S4D and E, ESI†). Glutathione ethyl ester (a cell-permeable reducing agent), *N*-acetylcysteine (a GSH biosynthesis raw material), ferrostatin-1 (a ferroptosis inhibitor), and deferoxamine mesylate (an iron chelator) were able to restore cell viability after TDI-COF-enhanced radiotherapy, irrespective of the X-ray dose (4 or 8 Gy), whereas Z-VAD-FMK (an apoptosis inhibitor), necrostatin-1s (a necroptosis inhibitor), or 3-methyladenine (an autophagy inhibitor) did not, which fully supports a ferroptosis mechanism.^91^

In addition to colorectal cancer, TDI-COF also enhanced radiotherapy for breast cancer (Fig. S5A and B, ESI†), even at very low X-ray doses (*e.g.*, 2 Gy). Experiments have shown that MCF-7 cell death involves ferroptosis (but other cell death mechanisms cannot be completely excluded) and is closely associated with oxidative stress caused by DNA damage and GSH depletion (Fig. S5C–G, ESI†).

The tumor retention time of radiosensitizers is critical for enhancing X-ray deposition at the tumor sites.^92^ The tumor retention with a half-life of approximately 4 h after intratumoral injection of TDI-COF into HCT-116 tumor-bearing nude mice was significantly higher than that of iopromide (<2 h), which may be related to the enhanced permeability and retention (EPR) effect (Fig. S6A, ESI†).^93^TDI-COF was primarily excreted through urine 72 h after the injection (Fig. S6B, ESI†), with an excretion rate of 64.9 ± 10.4%, which was comparable to that of iopromide (78.6 ± 13.4%) but obviously higher than that of UiO-66(Hf) (30.2 ± 15.4%). As a result, TDI-COF has acceptable intratumoral retention and low residue *in vivo*, resulting in minimum potential toxicity while assuring the effectiveness of tumor radiotherapy.

**Fig. 4:**
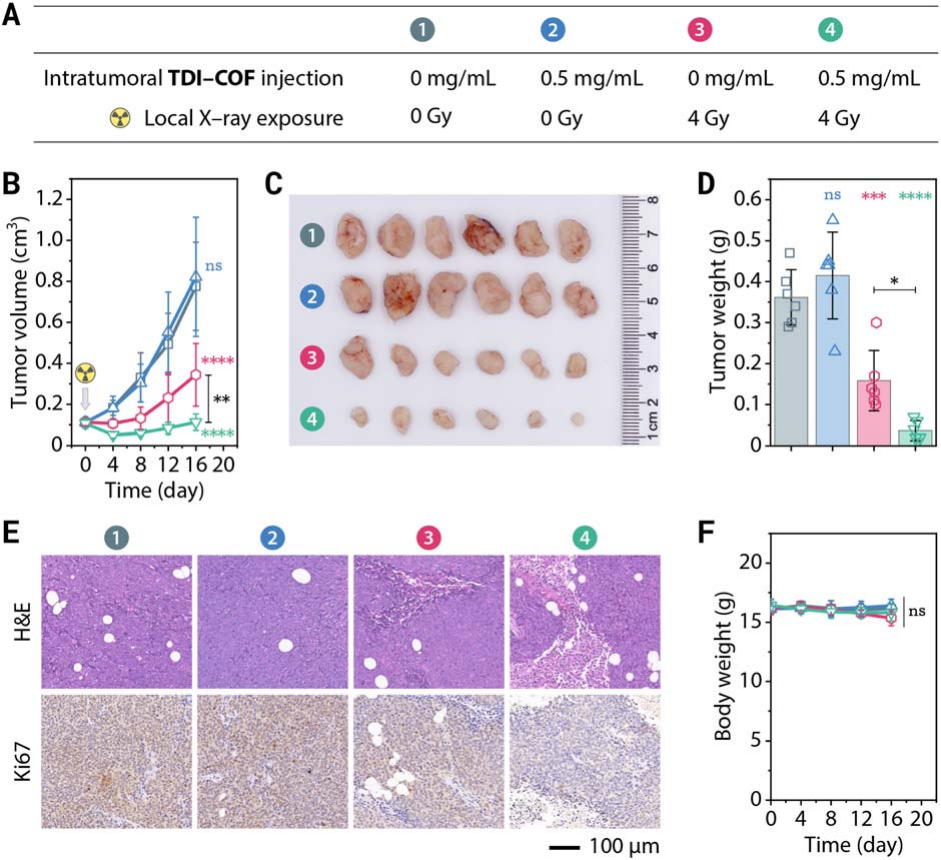
TDI-COF-enhanced radiotherapy in HCT-116 tumor-bearing nude mice. (A) Therapeutic schedule. (B) Tumor growth curves. (C) Weights of tumor tissue obtained by dissection. (D) Photographic images of the obtained tumors at the treatment endpoint. (E) Representative H&E and Ki67 staining images of the tumor tissue sampled at the treatment endpoint. (F) Body weight curves. Data are presented as means ± SDs (*n* = 6) and compared by two-way ANOVA followed by Tukey's *post hoc* test (B, F) and one-way ANOVA followed by Tukey's *post hoc* test (D). ****p < 0.0001; ***p < 0.001; **p < 0.01; *p < 0.05; *ns*, no significance (*p* > 0.05).

Encouraged by the remarkable efficacy of TDI-COF, its good *in vitro* biocompatibility and *in vivo* long tumor retention, we next examined its efficacy in *in vivo* radiotherapy (Fig. 4A). Tumors injected intratumorally with TDI-COF and exposed to low doses of X-rays were almost completely suppressed after 16 days, while tumors treated with X-rays alone showed only slightly lower growth rates (Fig. 4B–D). Tumors pathologically analyzed following treatment exhibited trends consistent with the tumor growth curves (Fig. 4E). Hematoxylin–eosin (H&E) staining revealed that a high percentage of cells in the group treated with TDI-COF and exposed to X-rays exhibited ferroptosis- and necrosis-like damage characterized by karyorrhexis, karyopyknosis, and ruptured plasma membranes.^94^ Immunohistochemical staining showed that the level of Ki67, a proliferative and prognostic marker,^95^ was consistently significantly lower in the group receiving TDI-COF-enhanced radiotherapy than in the group receiving radiotherapy alone. In addition, the almost no weight loss observed during radiotherapy suggests that TDI-COF is biosafe (Fig. 4F). Histological analysis of H&E stains of the major organs obtained at the treatment endpoint showed that there was no obvious abnormality of pathological observation, further supporting the biocompatibility of TDI-COF (Fig. S7, ESI†). In brief, these preliminary results underscore the *in vivo* effectiveness and biocompatibility of the TDI-COF radiosensitizer.

## Conclusions

In conclusion, we synthesized an iodide-containing and iminium-linked COF by a three-component one-pot *in situ* reaction at room temperature. The generated TDI-COF significantly improved X-ray deposition in colorectal cancer cells, effectively sensitized low-dose X-ray-induced radiotherapy, triggered ferroptotic cell death by damaging DNA and promoting lipid peroxidation, and displayed potent *in vitro* and *in vivo* antitumor activities as a consequence. Although further laboratorial and preclinical experiments including targeted delivery, catabolic pathways, and genotoxicity are necessary, our study demonstrated that metal-free COFs can be potential sensitizers for enhancing radiotherapy and possibly provide a new method for developing COF-based oncotherapies.

## Author contributions

Conceptualization, Y.-B. Dong. Investigation, L.-L. Zhou, Q. Guan, and W. Zhou. Software, J.-L. Kan. Methodology, L.-L. Zhou, Q. Guan, and Y.-B. Dong. Project administration, Y.-B. Dong. Resources, Y.-B. Dong. Supervision, Y.-B. Dong. Visualization, Q. Guan. Writing – original draft, L.-L. Zhou and Q. Guan. Writing – review & editing, L.-L. Zhou, Q. Guan, and Y.-B. Dong.

## Data availability

The data that support the findings of this study are presented in the paper and the ESI.†

## Conflicts of interest

There are no conflicts to declare.

## Supplementary Material

SC-014-D3SC00251A-s001
