# Supplementary material for: An iodide-containing covalent organic framework for enhanced radiotherapy
Source: Chem Sci. 2023 Mar 6;14(13):3642–51. doi: 10.1039/d3sc00251a (PMC10056114; doi:10.1039/d3sc00251a)
Supplement: SC-014-D3SC00251A-s001 [file SC-014-D3SC00251A-s001.pdf]

Electronic Supplementary Material (ESI) for Chemical Science.
This journal is © The Royal Society of Chemistry 2023

Chem. Sci., **2023**. https://doi.org/10.1039/D3SC00251A

Electronic supplementary information

An iodide-containing covalent organic framework for enhanced radiotherapy

Le-Le Zhou,[a,‡] Qun Guan,[a,‡] Wei Zhou,[b] Jing-Lan Kan,[a] and Yu-Bin Dong[a,*]

a. College of Chemistry, Chemical Engineering and Materials Science, Collaborative Innovation Center of Functionalized Probes for Chemical Imaging in Universities of Shandong, Key Laboratory of Molecular and Nano Probes, Ministry of Education, Shandong Normal University, Jinan 250014, China. *E-mail: yubindong@sdnu.edu.cn (Y.-B. Dong)

b. Department of Oncology, Shandong Provincial Hospital Affiliated to Shandong First Medical University, Jinan 250021, China.

‡ These authors contributed equally.

Table S1 Atomic coordinates of the AA-stacking mode of **TDI-COF**.

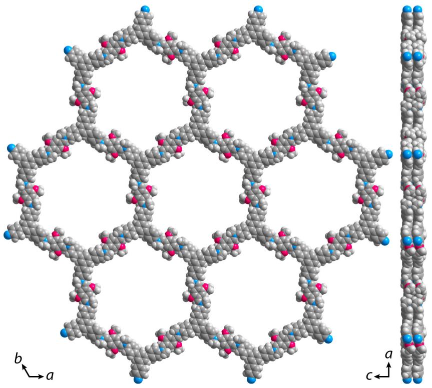

Space group: *P*3 (143). $a = b = 37.4508$ Å, $c = 3.4566$ Å, $\alpha = \beta = 90°$, $\gamma = 120°$

	x	*y*	*z*		*x*	*y*	*z*
**C1**	1.45667	−0.52564	0.38862	**C30**	1.42475	−0.60998	0.18634
**C2**	1.48134	−0.54277	0.37185	**C31**	1.57514	−0.39013	0.18590
**C3**	1.52614	−0.51879	0.41057	**C32**	1.61721	−0.48489	0.63444
**C4**	1.54324	−0.47444	0.38835	**H33**	1.42342	−0.54567	0.36352
**C5**	1.51858	−0.45732	0.37162	**H34**	1.57646	−0.45444	0.36304
**C6**	1.47376	−0.48133	0.41061	**H35**	1.47208	−0.42611	0.41532
**C7**	1.45316	−0.45958	0.44859	**H36**	1.28534	−0.40888	0.50831
**C8**	1.30652	−0.37560	0.50854	**H37**	1.30894	−0.44323	0.31737
**C9**	1.34906	−0.36061	0.50863	**H38**	1.33502	−0.49020	0.32750
**C10**	1.36566	−0.38948	0.50918	**H39**	1.45426	−0.39105	0.70198
**C11**	1.34087	−0.43115	0.40951	**H40**	1.42736	−0.34466	0.70371
**C12**	1.35580	−0.45823	0.41396	**H41**	1.39824	−0.52711	0.81724
**C13**	1.39706	−0.44477	0.51252	**H42**	1.36843	−0.53680	0.39044
**C14**	1.42232	−0.40298	0.61015	**H43**	1.35755	−0.51590	0.80286
**C15**	1.40670	−0.37643	0.61069	**H44**	1.52783	−0.57398	0.41541
**N16**	1.41166	−0.47306	0.51599	**H45**	1.54569	−0.60904	0.70147
**C17**	1.38269	−0.51520	0.63471	**H46**	1.57262	−0.65540	0.70308
**C18**	1.54674	−0.54051	0.44851	**H47**	1.69102	−0.55679	0.31691
**N19**	1.58825	−0.52704	0.51571	**H48**	1.66491	−0.50984	0.32716
**C20**	1.60287	−0.55529	0.51216	**H49**	1.59112	−0.69426	0.50781
**C21**	1.57762	−0.59711	0.60971	**H50**	1.42278	−0.63779	0.05854
**C22**	1.59325	−0.62362	0.61015	**H51**	1.41683	−0.59386	−0.03385
**C23**	1.63430	−0.61059	0.50871	**H52**	1.40115	−0.62006	0.41819
**C24**	1.65908	−0.56888	0.40904	**H53**	1.57712	−0.36232	0.05811
**C25**	1.64412	−0.54182	0.41362	**H54**	1.58306	−0.40625	−0.03429
**C26**	1.65093	−0.63941	0.50810	**H55**	1.59874	−0.38005	0.41776
**C27**	1.62440	−0.68213	0.50802	**H56**	1.63147	−0.46328	0.39016
**O28**	1.53414	−0.41509	0.32837	**H57**	1.64236	−0.48417	0.80263
**O29**	1.46574	−0.58502	0.32875	**H58**	1.60166	−0.47298	0.81698

Table S2 Atomic coordinates of the AB-stacking mode of **TDI-COF**.

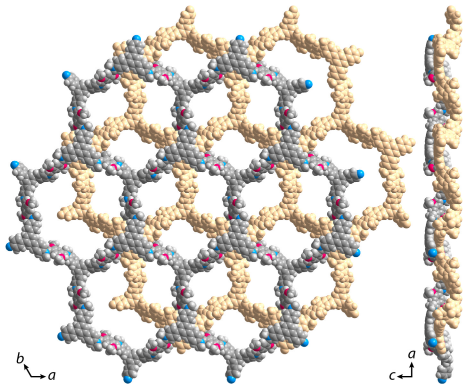

Space group: $P6_3$ (173). $a = b = 35.2753$ Å, $c = 6.1093$ Å, $\alpha = \beta = 90°$, $\gamma = 120°$

	x	y	z		x	y	z
**C1**	1.13300	−0.65859	0.09340	**C30**	1.06795	−0.73335	−0.14213
**C2**	1.12192	−0.70070	0.14971	**C31**	1.23184	−0.59796	0.72298
**C3**	1.14074	−0.71062	0.34016	**C32**	1.19417	−0.74684	0.58712
**C4**	1.16920	−0.67322	0.47872	**H33**	1.12087	−0.65293	−0.05899
**C5**	1.17865	−0.63137	0.42806	**H34**	1.18064	−0.67940	0.63229
**C6**	1.16264	−0.62092	0.22765	**H35**	1.20131	−0.55400	0.30118
**C7**	1.17833	−0.57694	0.18135	**H36**	1.25329	−0.35995	−0.20794
**C8**	1.28834	−0.34824	−0.21343	**H37**	1.22462	−0.43279	−0.43241
**C9**	1.30287	−0.37891	−0.21149	**H38**	1.16957	−0.50718	−0.34439
**C10**	1.27053	−0.42655	−0.17880	**H39**	1.25429	−0.50894	0.16988
**C11**	1.23089	−0.44894	−0.29640	**H40**	1.30800	−0.43472	0.08068
**C12**	1.19906	−0.49179	−0.24443	**H41**	1.10411	−0.60257	−0.01192
**C13**	1.20490	−0.51450	−0.06686	**H42**	1.12965	−0.59046	−0.27383
**C14**	1.24623	−0.49296	0.04055	**H43**	1.11841	−0.55262	−0.13804
**C15**	1.27765	−0.45023	−0.01322	**H44**	1.10431	−0.77868	0.27152
**N16**	1.17172	−0.55660	−0.00207	**H45**	1.06204	−0.80993	0.59486
**C17**	1.12915	−0.57664	−0.11277	**H46**	1.01233	−0.88572	0.67667
**C18**	1.13005	−0.75381	0.37534	**H47**	1.12153	−0.90969	0.69060
**N19**	1.14800	−0.77110	0.52332	**H48**	1.17127	−0.83398	0.61918
**C20**	1.12096	−0.81512	0.59089	**H49**	0.97240	−0.94768	0.70015
**C21**	1.07539	−0.83166	0.61473	**H50**	1.04465	−0.76675	−0.19563
**C22**	1.04689	−0.87549	0.66218	**H51**	1.08819	−0.71502	−0.28366
**C23**	1.06159	−0.90599	0.68475	**H52**	1.04909	−0.71784	−0.08661
**C24**	1.10744	−0.88857	0.67249	**H53**	1.25407	−0.60861	0.65570
**C25**	1.13658	−0.84450	0.63040	**H54**	1.21286	−0.62008	0.85783
**C26**	1.03013	−0.95406	0.70360	**H55**	1.25161	−0.56486	0.78937
**C27**	0.98452	−0.97057	0.70398	**H56**	1.21277	−0.71937	0.47645
**O28**	1.20466	−0.59577	0.55760	**H57**	1.20915	−0.76759	0.57965
**O29**	1.09319	−0.73677	0.02953	**H58**	1.19869	−0.73408	0.75564

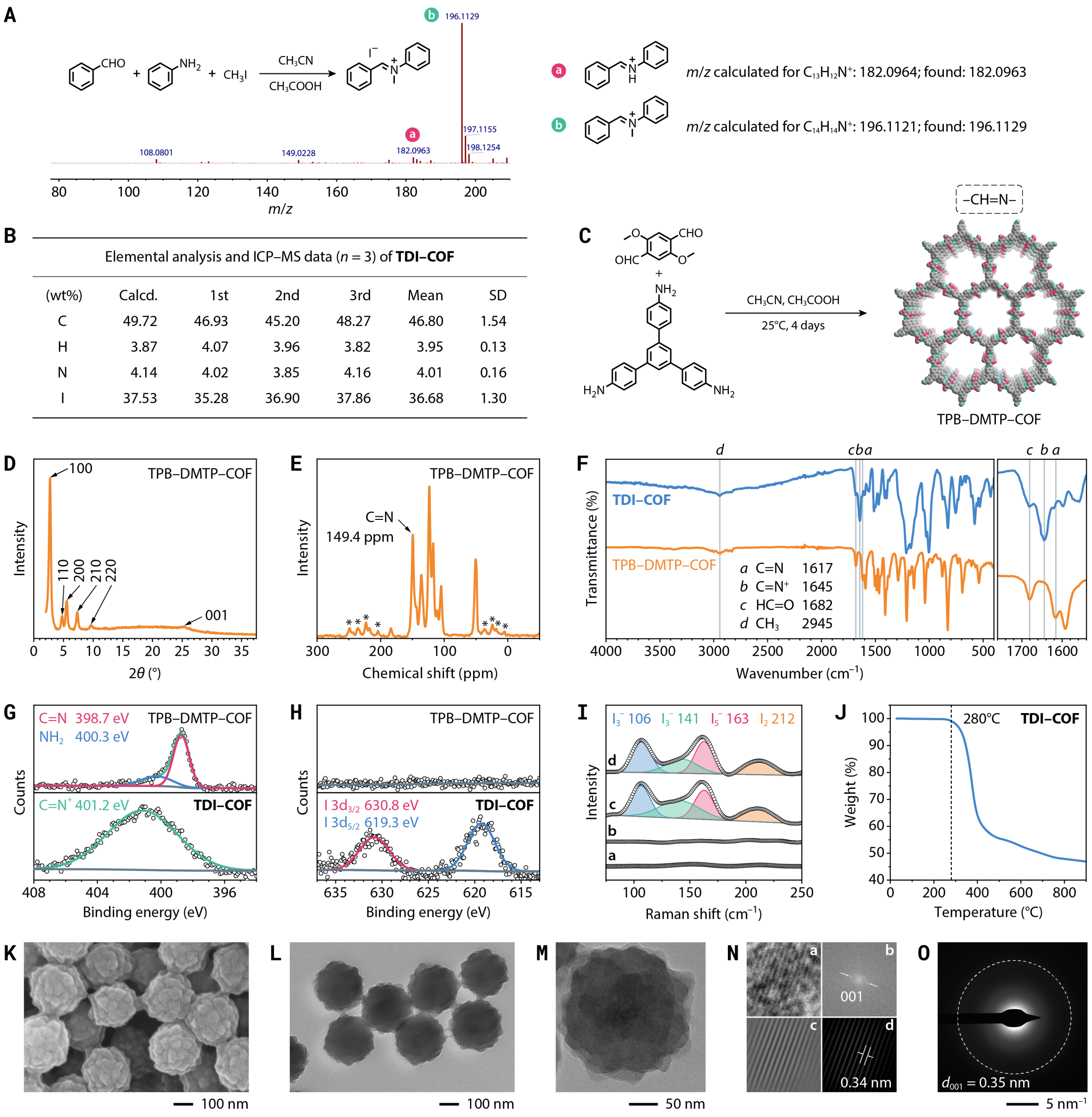

Elemental analysis and ICP–MS data (n = 3) of **TDI–COF**

(wt%)	Calcd.	1st	2nd	3rd	Mean	SD
C	49.72	46.93	45.20	48.27	46.80	1.54
H	3.87	4.07	3.96	3.82	3.95	0.13
N	4.14	4.02	3.85	4.16	4.01	0.16
I	37.53	35.28	36.90	37.86	36.68	1.30

Fig. S1 Characterization of **TDI-COF** and TPB-DMTP-COF.

(A) High-resolution mass spectrum of *N*-benzylidene-*N*-methylbenzenaminium ion.

(B) Elemental analysis and ICP–MS data of **TDI-COF**.

(C) Synthesis of unmethylated imine-linked TPB-DMTP-COF.

(D) PXRD pattern of TPB-DMTP-COF.

(E) ^{13}C CP–MAS NMR spectrum of TPB-DMTP-COF. *Spinning side bands.

(F) ATR FT-IR spectra of **TDI-COF** and TPB-DMTP-COF. Inset: enlarged view in the range of 1540–1760 cm^{-1}.

(G) High-resolution XPS spectra of **TDI-COF** and TPB-DMTP-COF in the N 1s region.

(H) High-resolution XPS spectra of **TDI-COF** and TPB-DMTP-COF in the I 3d region.

(I) Raman spectra of TPB-DMTP-COF (a), **TDI-COF** (b), **I$_2$@TPB-DMTP-COF** (c) and **I$_2$@TDI-COF** (d).

(J) TGA curve of **TDI-COF**.

(K) SEM image of **TDI-COF**.

(L) TEM image of **TDI-COF**.

(M) HRTEM images of **TDI-COF**.

(N) Lattice-resolution HRTEM image of **TDI-COF** (a), selected area FFT pattern (b), and the corresponding bandpass-filtered images with enhanced contrast (c, d).

(O) Selected area electron diffraction pattern of **TDI-COF**.

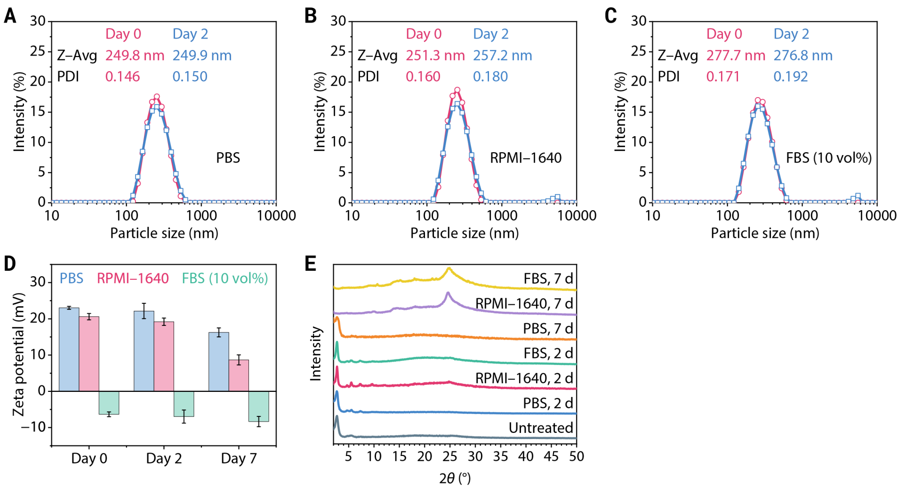

Fig. S2 Stability tests of **TDI-COF**.

(A) Time-dependent DLS measurements of **TDI-COF** dispersed in PBS.

(B) Time-dependent DLS measurements of **TDI-COF** dispersed in RPMI-1640.

(C) Time-dependent DLS measurements of **TDI-COF** dispersed in FBS (10 vol%).

(D) Zeta potentials of **TDI-COF** after dispersion in PBS, RPMI-1640, and FBS (10 vol%) for 2 or 7 days.

(E) PXRD patterns of **TDI-COF** after dispersion in PBS, RPMI-1640, and FBS (10 vol%) for 2 or 7 days.

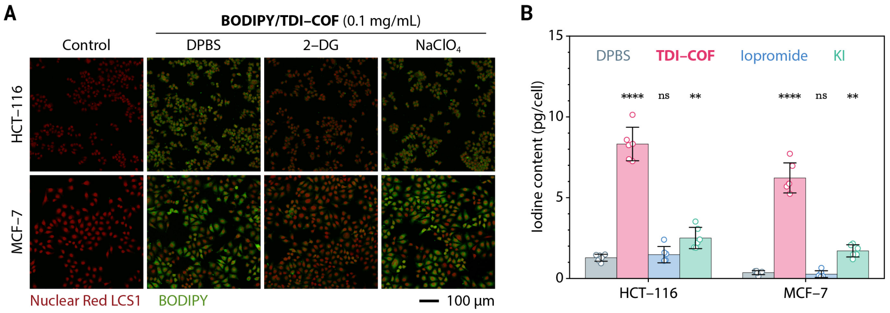

Fig. S3 Cellular uptake of **TDI-COF** in the presence of 2-DG as a glycolysis inhibitor and $NaClO_4$ as a competitive inhibitor of the sodium–iodide symporter.

(A) Confocal laser scanning microscopic images of HCT-116 and MCF-7 cells incubated with 2-DG (0 or 150 mM) and $NaClO_4$ (0 or 0.1 mM, 1 h) before incubation of **BODIPY/TDI-COF** (0 or 0.1 mg/mL, 4 h).

(B) Intracellular iodine contents of HCT-116 and MCF-7 cells treated with **TDI-COF** (10 mL, 0.2 mg/mL), iopromide (10 mL, 0.15 mg/mL), and KI (10 mL, 0.096 mg/mL) for 2 h. Data are presented as means ± SDs ($n = 6$) and compared by two-way ANOVA followed by Dunnett's multiple comparison test. ****$p < 0.0001$; **$p < 0.01$; *ns*, no significance ($p > 0.05$).

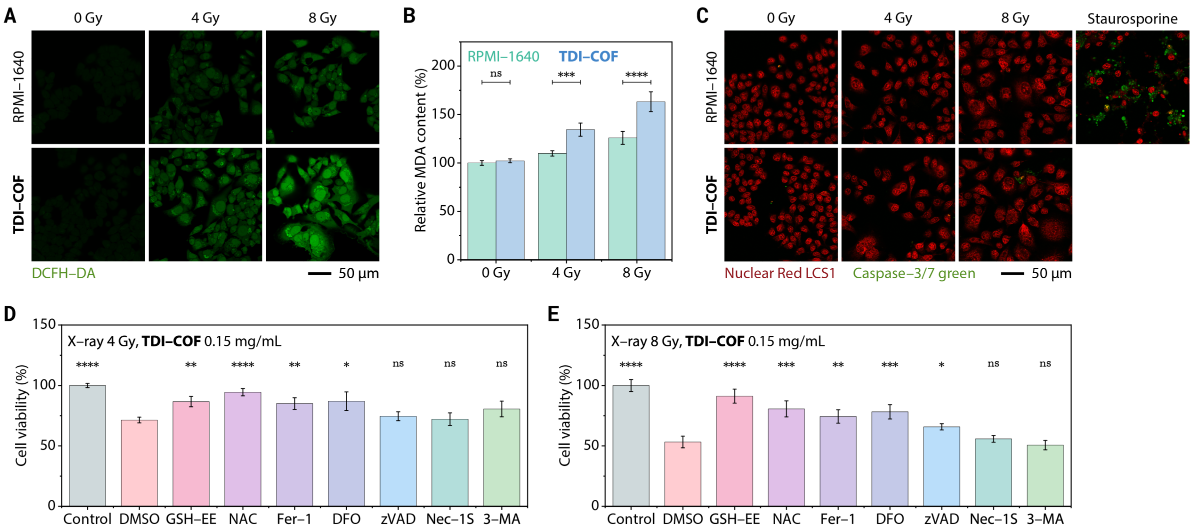

Fig. S4 Ferroptosis-related radiotherapy of HCT-116 cells pretreated with **TDI-COF** (0 or 0.15 mg/mL) for 4 h and exposed to X-ray radiation (0, 4, or 8 Gy).

(A) Confocal laser scanning microscopic images of intracellular ROS detection using the DCFH-DA fluorescence probe.

(B) Intracellular MDA levels.

(C) Caspase 3 activation assays. Staurosporine-induced apoptotic cells were used as a positive control.

(D) Cell death rescue experiments of HCT-116 cells exposed to X-ray radiation (4 Gy).

(E) Cell death rescue experiments of HCT-116 cells exposed to X-ray radiation (8 Gy).

Data are presented as the means ± SDs, n = 3 (B) or 5 (D, E). Data are compared by two-way ANOVA followed by Bonferroni's multiple comparison test (B) and Welch's ANOVA followed by Dunnett's T3 *post hoc* test (D, E). ****$p < 0.0001$; ***$p < 0.001$; **$p < 0.01$; *$p < 0.05$; *ns*, no significance ($p > 0.05$).

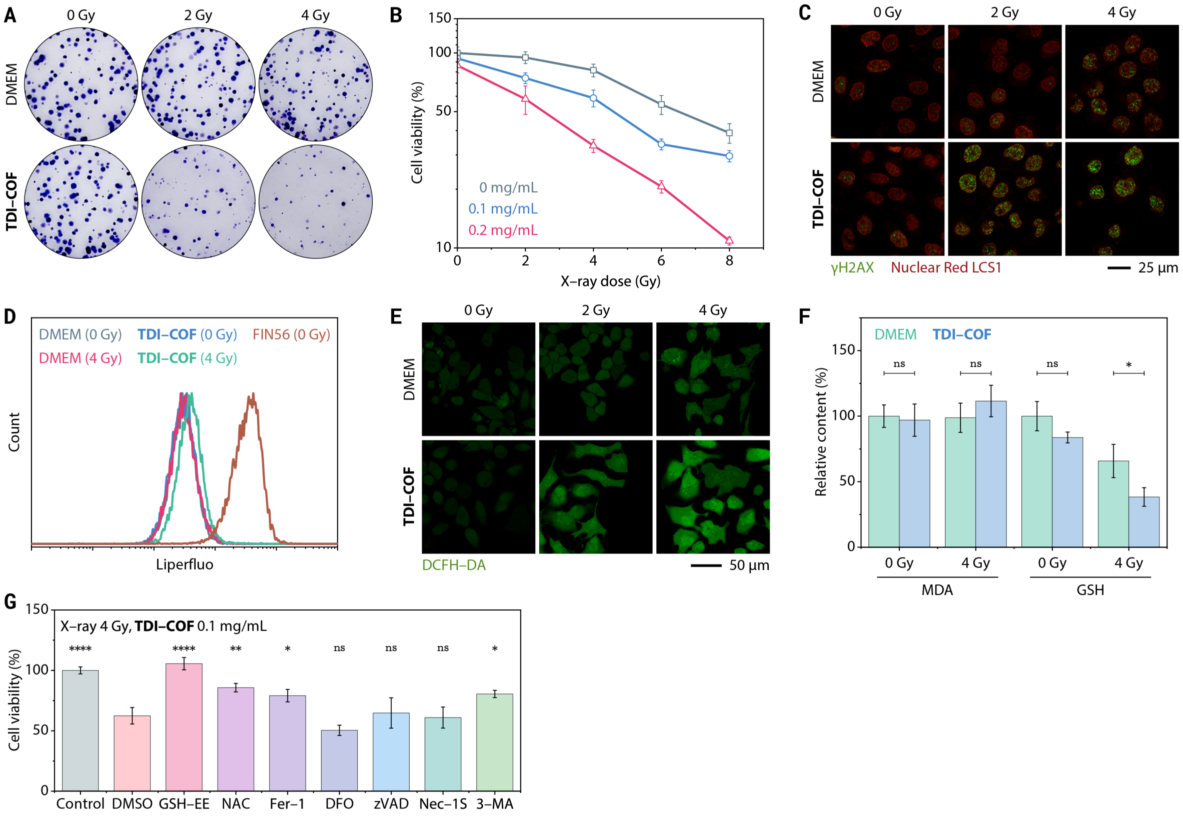

Fig. S5 Oxidative stress-related radiotherapy of MCF-7 cells pretreated with **TDI-COF** (0–0.2 mg/mL) for 4 h and exposed to X-ray radiation (0–8 Gy).

(A) Clonogenic assay.

(B) CCK-8 cell viability assay.

(C) Confocal laser scanning microscopy images of γH2AX immunofluorescence staining.

(D) Flow cytometric analysis of intracellular lipid peroxides. FIN56-induced ferroptotic cells were used as a positive control.

(E) Confocal laser scanning microscopic images of intracellular ROS detection using the DCFH-DA fluorescence probe.

(F) Intracellular MDA and GSH levels.

(G) Cell death rescue experiments of MCF-7 cells.

Data are presented as the means ± SDs ($n = 3$) and compared by two-way ANOVA followed by Šídák's multiple comparison test (F) and one-way ANOVA followed by Dunnett's *post hoc* test (G). ****$p < 0.0001$; ***$p < 0.001$; **$p < 0.01$; *$p < 0.05$; *ns*, no significance ($p > 0.05$).

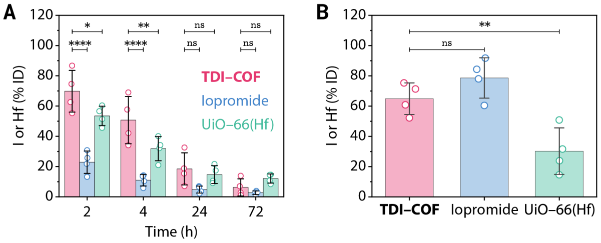

Fig. S6 Tumor retention of **TDI-COF** (0.5 mg/mL), iopromide (0.38 mg/mL), and UiO-66(Hf) (0.57 mg/mL) in HCT-116 tumor-bearing nude mice.

(A) I and Hf contents in the tumor at different times after the intratumoral injection.

(B) I and Hf contents in the urine 72 h after the intratumor injection.

Data are presented as means ± SDs (n = 4) and compared by two-way ANOVA followed by Tukey's multiple comparison test (A) and one-way ANOVA followed by Dunnett's multiple comparison test (B). ****$p < 0.0001$; **$p < 0.01$; *$p < 0.05$; *ns*, no significance ($p > 0.05$).

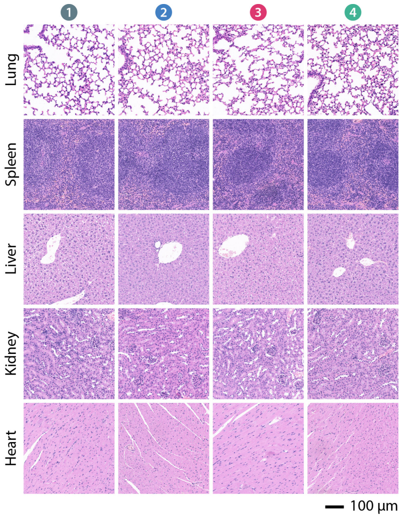

Fig. S7 H&E staining of the main organs collected at the end of the treatment.

Experimental materials

Methanol, ethanol, acetic acid, *N*,*N*-dimethylformamide (DMF), and acetonitrile were purchased from Sinopharm (Beijing, China).

1,3,5-Tris(4-aminophenyl)benzene and 2,5-dimethoxyterephthalaldehyde were purchased from Jilin Chinese Academy of Sciences – Yanshen Technology (Changchun, China).

Iodomethane was purchased from Xiya Reagent (Linyi, China).

Dimethyl sulfoxide (DMSO), glutathione ethyl ester (GSH-EE) and poly(2-hydroxyethyl methacrylate) were purchased from Sigma–Aldrich (Shanghai, China).

Iohexol and potassium iodide were purchased from Aladdin (Shanghai, China).

Iopromide injection (Ultravist 300) was purchased from Bayer (Leverkusen, Germany).

Hafnium(IV) chloride, I_2, and 5,5-difluoro-1,3,7,9-tetramethyl-10-phenyl-5*H*-dipyrrolo[1,2-*c*:2',1'-*f*][1,3,2]diazaborinin-4-ium-5-uide (BODIPY) were purchased from Macklin (Shanghai, China).

Terephthalic acid was purchased from Shanghai Haohong Scientific Co.,Ltd.

Tween-20, Triton X-100, 3-methyladenine (3-MA), and *N*-acetylcysteine (NAC) were purchased from TCI (Shanghai, China).

Hoechst 33342 trihydrochloride, FIN56, staurosporine, 2',7'-dichlorodihydrofluorescein diacetate (DCFH-DA), ferrostatin-1 (Fer-1), necrostatin-1s (Nec-1S), deferoxamine mesylate (DFO), 2-deoxy-*D*-glucose (2-DG), protease inhibitor cocktail (Cat# HY-K0010), and phosphatase inhibitor cocktail (Cat# HY-K0022) were purchased from MedChemExpress (Shanghai, China).

A cell counting kit-8 (CCK-8, Cat# CK04), Liperfluo (Cat# L248), and Lipi-Blue (Cat# LD01) were purchased from Dojindo (Beijing, China).

Nuclear Red LCS1 was purchased from AAT Bioquest, Inc.

A reduced GSH assay kit and a hematoxylin–eosin (H&E) stain kit were purchased from Nanjing Jiancheng Bioengineering Institute.

A malondialdehyde (MDA) assay kit was purchased from Abbkine (Wuhan, China).

Roswell Park Memorial Institute 1640 medium (RPMI-1640, Cat# C11875500CP), Dulbecco's modified eagle medium (DMEM, Cat# C11995500CP), Hank's balanced salt solution (HBSS, Cat# C14175500BT), GlutaMAX (Cat# 35050061), CellEvent Caspase-3/7 Green (Cat# C10423), Alexa Fluor 633-conjugated goat anti-rabbit IgG (H+L) cross-adsorbed secondary antibody (Cat# A-21070), Alexa Fluor 488-conjugated goat anti-rabbit IgG (H+L) cross-adsorbed secondary antibody (Cat# A-11008), BCA protein assay kit (Cat# 23227), and PageRuler prestained protein ladder (10–180 kDa, Cat# 26616) were purchased from Thermo Fisher Scientific Inc.

Z-VAD-FMK (zVAD) was purchased from Selleck Chemicals LLC.

RIPA lysis buffer (Cat# K1120) was purchased from APExBIO (Houston, USA).

Trypsin (0.25 wt%) and ethylenediaminetetraacetic acid (EDTA, 0.02 wt%) in Puck's saline A (trypsin/EDTA solution) was purchased from Biological Industries (Sartorius Group, USA).

Rabbit anti-ACSL4 (Cat# ab155282) and rabbit anti-GPX4 (Cat# ab125066) were purchased from Abcam (Shanghai, China).

Horseradish peroxidase (HRP)-conjugated goat anti-rabbit IgG (H+L) secondary antibody (Cat# SA00001-2) were purchased from Proteintech (Wuhan, China).

Rabbit anti-SLC7A11 (Cat# 12691) and rabbit anti-γH2AX (Ser139) (Cat# 9718) were purchased from Cell Signaling Technology, Inc.

Rabbit anti-β-tubulin (Cat# ET1602-4) was purchased from HuaBio (Hangzhou, China).

Tris-buffered saline powder (Cat# G0001-2L), SDS-PAGE running buffer powder (Cat# G2018-1L), PAGE transfer buffer powder (Cat# G2017-1L), and rabbit anti-Ki67 (Cat# GB111499) were purchased from Servicebio (Wuhan, China).

Fetal bovine serum (FBS), goat serum, 2-[4-(2-hydroxyethyl)-1-piperazinyl]ethanesulfonic acid (HEPES) solution, and phosphate-buffered saline (PBS) were purchased from VivaCell (Shanghai, China).

Normocin (Cat# ant-nr-2) was purchased from Invivogen (San Diego, USA).

SDS-PAGE sample loading buffer and Tris-HCl buffer (pH=7.4) were purchased from Beyotime (Shanghai, China).

A polyacrylamide gel fast preparation kit (Cat# PG112) was purchased from Epizyme (Shanghai, China).

A chemiluminescence detection kit (Cat# E412-01) was purchased from Vazyme (Nanjing, China).

Paraformaldehyde fixing solution was purchased from Babio (Jinan, China).

Tris-buffered saline with Tween-20 (TBST) and Giemsa staining solution (10×) were purchased from Solarbio (Beijing, China).

A polymer-enhanced two-step immunohistochemistry detection kit (Cat# PV-9001) and a diaminobenzidine kit (Cat# ZLI-9017) were purchased from ZSGB-BIO (Beijing, China).

Experimental instrumentation

Supercritical carbon dioxide drying was performed using a Tousimis Samdri-PVT-3D critical point dryer. Damp solids were contained in folded filter paper secured with a staple and dried with a 15 min purge time and 15 min equilibration time after heating.

Inductively coupled plasma–mass spectrometry (ICP–MS) measurements were carried out using a PerkinElmer NexION 300X ICP–MS.

Powder X-ray diffraction (PXRD) patterns were obtained on a SmartLab SE X-ray powder diffractometer (Rigaku, Japan) with Cu Kα line focused radiation (λ = 1.54056 Å) in the range of 2θ = 2.00°–50.00° at a step size of 0.01° in a continuous mode. Powder samples were placed directly on zero-background sample holders and flattened using glass microscope slides.

Samples were degassed under vacuum at 120°C for 8 h, and nitrogen-adsorption isotherms were measured at 77 K using a Micromeritics ASAP2020 HD88 surface area and porosity analyzer. The Brunauer–Emmett–Teller (BET) equation was used to calculate the specific surface areas. The pore size distribution was derived from the sorption curve using the non-local density functional theory model.

^{13}C Cross-polarization magic-angle spinning (MAS) solid-state nuclear magnetic resonance (NMR) spectra were recorded on a Bruker Avance III HD 400 MHz wide-bore NMR spectrometer with a 4 mm double-resonance MAS probe.

Fourier transform infrared (FT-IR) spectra were obtained on a Thermo Scientific Nicolet iS50 FT-IR spectrometer equipped with a diamond attenuated total reflection (ATR) module between 4000–400 cm^{-1}. Each spectrum represented an average of 16 scans.

Mass spectrometry analysis was performed on a Bruker maXis Ultrahigh-resolution time-of-flight triple quadrupole mass spectrometer equipped with an electron spray ionization (ESI) source. All data were reported as mass per charge ratios (m/z).

Transmission electron microscopy (TEM) images were recorded on a Hitachi HT7700 120 kV compact-digital instrument. High-angle annular dark-field scanning transmission electron microscopy (HAADF–STEM) and elemental mapping images were recorded on a FEI Talos F200X high-resolution scanning transmission electron microscope. The samples were prepared in methanol by sonicating the material for 5 min, followed by application on a carbon-coated copper TEM grid (200 mesh), and air-dried at room temperature. The fast Fourier transform (FTT) and its inverse transform of the selected region were performed by ImageJ (version 1.53k) software.

Scanning electron microscopy (SEM) images were recorded using a Hitachi SU8010 instrument. The SEM samples were prepared by depositing a diluted suspension onto silicon wafers (approximately 3 × 3 mm), followed by air drying and coating with a thin layer of Pt to increase the contrast.

Elemental analyses were performed on an Elementar Vario EL Cube CHNS elemental analyzer.

Thermogravimetric analysis (TGA) was performed with a Mettler Toledo TGA/DSC3+ thermogravimetric analyzer. Approximately 4.0 mg of dried sample was analyzed from room temperature to 1000°C at a heating rate of 10°C/min under a N_2 atmosphere.

Raman spectrum was obtained by a LabRAM HR Evolution Raman spectrometer with a 532 nm laser.

The hydrodynamic particle size was measured using a Malvern Zetasizer Nano ZS90 system.

X-ray photoelectron spectroscopy (XPS) profiles were obtained on a Thermo Fisher Scientific ESCALAB 250Xi XPS System equipped with a monochromatic Al Kα radiation source (1486.6 eV). The C 1s signal located at 284.8 eV from adventitious carbon was used as binding energy reference. The Shirely baseline was subtracted from the overall signal data before data deconvolution.

Flow cytometric analysis was performed using a BD FACSCalibur flow cytometer equipped with an argon ion laser (488 nm) for excitation.

Microplate assays were conducted using a Molecular Devices SpectraMax i3x multimode microplate detection system.

Western blot images were obtained using GE Healthcare Amersham Imager 600/680 luminescent image analyzers.

Laser scanning confocal fluorescence images of cells were captured with a Leica TCS SP8 confocal laser scanning microscope equipped with 405 nm, 458 nm, 488 nm, 514 nm, 561 nm, and 633 nm lasers. The imaging scan speed was 400 Hz, and transmitted light was used to find the areas of interest to reduce photodamage to the sample. Glass bottom dishes and 4/8-well chamber slides (Cellvis, USA) were used for cell culture in experiments. The original culture media and PBS were replaced with HBSS supplemented with HEPES (25 mM, pH 7.4) and GlutaMAX (1×) to provide better buffering capacity under normal CO_2 concentrations before live cell imaging.

Computerized tomography (CT) was performed on a Siemens Somatom Force dual source CT scanner (Siemens Healthcare, Erlangen, Germany) with a tube voltage of 70 kV. Samples dispersed in PBS were added into polypropylene centrifuge tubes that placed on a plexiglass shelf before CT scans. Multi-planar reformation images were generated using the reconstructed algorithm with strength 5, the Qr40 kernel, and a slice thickness of 0.5 mm.

X-ray radiation was carried out using a Varian Clinac 23EX linear accelerator at a dose rate of 4 Gy/min.

Cell culture

The HCT-116 (human colorectal carcinoma) cell line was provided by the Cell Bank, Chinese Academy of Sciences (Shanghai, China), and cultured in RPMI-1640 medium supplemented with fetal bovine serum (10 vol%) and normocin (100 μg/mL) in a water-jacketed CO_2 incubator with CO_2 (5 vol%) at 37°C.

The MCF-7 (human breast adenocarcinoma) cell line was provided by Institute of Basic Medicine, Shandong Academy of Medical Sciences (Jinan, China), and cultured in DMEM supplemented with fetal bovine serum (10 vol%), GlutaMAX (1×), human recombinant insulin (10 μg/mL), and normocin (100 μg/mL) in a water-jacketed CO_2 incubator with CO_2 (5 vol%) at 37°C.

Synthesis of TDI-COF

A mixture of 1,3,5-tris(4-aminophenyl)benzene (738 mg, 2.1 mmol), 2,5-dimethoxyterephthalaldehyde (612 mg, 3.15 mmol), iodomethane (100 mL), acetic acid (37.5 mL), and acetonitrile (750 mL) was stirred at 600 rpm for 4 days at 25°C. The precipitate was collected by centrifugation at 12000 rpm for 30 min at 4°C, washed three times with acetonitrile, and then washed three times with ethanol. Finally, the powders were dried in supercritical carbon dioxide. Yield: 1.27 g (60%).

Synthesis of TPB-DMTP-COF

2,5-Dimethoxyterephthalaldehyde (204 mg, 1.05 mmol) and 1,3,5-tris(4-aminophenyl)benzene (246 mg, 0.7 mmol) were charged in a wild-mouth bottle containing acetonitrile (250 mL). The mixture was added acetic acid (12.5 mL) and stirred at 600 rpm for 4 days at 25°C. The precipitate was collected by centrifugation at 12000 rpm for 30 min at 4°C, washed three times with acetonitrile, and then washed three times with ethanol. Finally, the powders were dried in supercritical carbon dioxide to afford yellow solid product.

Synthesis of UiO-66(Hf)

A mixture of $HfCl_4$ (320 mg, 1.0 mmol), terephthalic acid (166 mg, 1.0 mmol), acetic acid (4.0 mL), and water (6.0 mL) was stirred at 600 rpm for 24 h at 100°C. The precipitate was collected by centrifugation at 12000 rpm for 30 min at 4°C, washed three times with ethanol, and dried in supercritical carbon dioxide.

Synthesis of I_2@TDI-COF

TDI-COF (10 mg) was dispersed in iodine 1-hexane solution (10 mL, 1 mg/mL) and stirred at 300 rpm for 12 h at 25°C. The precipitate was collected by centrifugation at 12000 rpm for 30 min at 4°C, washed three times with ethanol, and dried in air at room temperature.

Synthesis of I_2@TPB-DMTP-COF

TPB-DMTP-COF (10 mg) was dispersed in iodine 1-hexane solution (10 mL, 1 mg/mL) and stirred at 300 rpm for 12 h at 25°C. The precipitate was collected by centrifugation at 12000 rpm for 30 min at 4°C, washed three times with ethanol, and dried in air at room temperature.

Synthesis of BODIPY/TDI-COF

TDI-COF (2 mg) was dispersed in BODIPY ethanol solution (5 mL, 150 μg/mL) and stirred at 500 rpm for 24 h at 25°C. The precipitate was collected by centrifugation at 12000 rpm for 30 min at 4°C, washed three times with ethanol, and dried in air at room temperature.

Mass spectrometry analysis

A mixture of aniline (100 μL, 1.1 mmol), benzaldehyde (100 μL, 1 mmol), iodomethane (500 μL, 8 mmol), acetic acid (20 μL), and acetonitrile (1.0 mL) was stirred at 200 rpm for 1 h at 25°C. The solution was diluted with acetonitrile for mass spectrometry analysis.

Cellular uptake

For visualizing cellular uptake, cells cultured in 4-well glass-bottom dishes were pretreated with 2-DG (400 μL, 150 mM) or $NaClO_4$ (400 μL, 0.1 mM) for 1 h in a CO_2 incubator. The pretreated and untreated cells were cultured with **BODIPY/TDI-COF** (400 μL, 0.1 mg/mL) for 4 h in a CO_2 incubator and washed twice with PBS. The cells were loaded with Nuclear Red LCS1 (200 μL, 1.0 μM) for 30 min in a CO_2 incubator. The cells were washed twice with PBS and laser scanning confocal fluorescence images were captured. The green images of **BODIPY/TDI-COF** were recorded at an excitation wavelength of 488 nm and an emission wavelength range of 500–540 nm. The red signal of Nuclear Red LCS1 was recorded at an excitation wavelength of 633 nm and an emission wavelength range of 640–690 nm. Untreated cells were used as the control group.

To further quantify the changes in intracellular iodine contents, cells cultured in T175 flasks were treated with **TDI-COF** (10 mL, 0.2 mg/mL), iopromide (10 mL, 0.15 mg/mL), and KI (10 mL, 0.096 mg/mL) for 2 h in a CO_2 incubator and carefully washed 4 times with PBS. The cells were collected and digested in aqua regia. The intracellular iodine contents were detected by ICP–MS.

Cell morphology and lipid droplet staining assays

HCT-116 cells were cultured in 4-well glass bottom dishes for 48 h, treated with **TDI-COF** (400 μL, 0 or 0.15 mg/mL) for 4 h in a CO_2 incubator, and exposed to X-ray radiation (0, 4, or 8 Gy). After an additional 48 h of incubation, the cells were loaded with Lipi-Blue (200 μL, 0.2 μM) for 15 min in a CO_2 incubator, followed by incubation with Nuclear Red LCS1 (200 μL, 1.0 μM) for 30 min in a CO_2 incubator. The cells were washed twice with PBS and laser scanning confocal fluorescence images were captured. The green signal of Lipi-Blue was recorded at an excitation wavelength of 405 nm and an emission wavelength range of 430–470 nm. The red signal of Nuclear Red LCS1 was recorded at an excitation wavelength of 633 nm and an emission wavelength range of 640–690 nm. Untreated cells were used as the control group.

CCK-8 cell viability assays

HCT-116 cells cultured in 96-well plates were treated with **TDI-COF** (100 μL, 0–0.25 mg/mL) for 4 h in a CO_2 incubator. The cells were exposed to exposed to X-ray radiation (0–8 Gy), and incubated in a CO_2 incubator for 48 h. Subsequently, the cells were washed three times with PBS. Then, RPMI-1640 (90 μL) and CCK-8 solution (10 μL) were added to each well, and the plate was incubated in a CO_2 incubator for approximately 1 h. The absorbance at 450 nm was measured using a multimode microplate detection system. Untreated cells were used as the control group, while cells treated with methanol for 1 h at 25°C were used as the blank.

MCF-7 cells cultured in 96-well plates were treated with **TDI-COF** (100 μL, 0–0.2 mg/mL) for 4 h in a CO_2 incubator. The cells were exposed to exposed to X-ray radiation (0–8 Gy), and incubated in a CO_2 incubator for 48 h. Subsequently, the cells were washed three times with PBS. Then, DMEM (90 μL) and CCK-8 solution (10 μL) were added to each well, and the plate was incubated in a CO_2 incubator for approximately 1 h. The absorbance at 450 nm was measured using a multimode microplate detection system. Untreated cells were used as the control group, while cells treated with methanol for 1 h at 25°C were used as the blank.

Clonogenic assays

HCT-116 cells were cultured at a density of 10^3 cells/well in 6-well plates for 24 h, treated with **TDI-COF** (2.0 mL, 0 or 0.15 mg/mL) for 4 h in a CO_2 incubator, and exposed to X-ray radiation (0 or 4 Gy). MCF-7 cells were cultured at a density of 10^3 cells/well in 6-well plates for 24 h, treated with **TDI-COF** (2.0 mL, 0 or 0.1 mg/mL) for 4 h in a CO_2 incubator, and exposed to X-ray radiation (0, 2, or 4 Gy). After approximately 7 days of incubation, the HCT-116 and MCF-7 cells were fixed with paraformaldehyde (2.0 mL, 4 wt%) for 24 h at 4°C and stained with fresh Giemsa staining solution for 6 h at room temperature. The plates were washed with water, air-dried, and photographed with a digital camera. The untreated well was used as the control group.

Sphere formation assays

To prepare poly(2-hydroxyethyl methacrylate)-coated 24-well plates, an ethanol solution of poly(2-hydroxyethyl methacrylate) (1.0 mL, 12 mg/mL) was added to each well of a 24-well plate, which was air-dried at room temperature and exposed to ultraviolet light for 24 h for further use.

HCT-116 cells were treated with **TDI-COF** (2.0 mL, 0 or 0.15 mg/mL) for 4 h and exposed to X-ray radiation (0 or 4 Gy). Subsequently, the cells were collected using a trypsin/EDTA solution, washed twice with PBS, and seeded into the coated 24-well plate. After approximately 10 days of incubation, the multicellular tumor spheroids were photographed with an inverted microscope. The untreated well was used as the control group.

γH2AX immunofluorescence staining assays

HCT-116 cells cultured in 4-well glass-bottom dishes were treated with **TDI-COF** (400 μL, 0 or 0.15 mg/mL) for 4 h and exposed to X-ray radiation (0, 4, or 8 Gy). After an additional 30 min of incubation, the cells were fixed in paraformaldehyde (400 μL, 4 wt%) for 24 h at 4°C and washed three times with PBS. The cells were permeabilized with Triton X-100 (400 μL, 0.5 vol%) for 5 min and washed twice with PBS. The cells were incubated in PBS (400 μL) containing normal goat serum (10 vol%) and Tween-20 (0.1 vol%)

for 1 h at room temperature. The cells were incubated with rabbit anti-γH2AX (Ser139) primary antibody (100 μL, 1:400 dilution, Cell Signaling Technology, Cat# 9718) at 4°C overnight and washed three times with PBS. The cells were incubated with Alexa Fluor 633-conjugated goat anti-rabbit IgG (H+L) cross-adsorbed secondary antibody (200 μL, 1:500 dilution, Thermo Fisher, Cat# A-21070) for 1 h at room temperature. Finally, the cell nuclei were counterstained with Hoechst 33342 (400 μL, 20 μM) for 30 min and washed twice with PBS. Laser scanning confocal fluorescence images were captured. The red images of Alexa Fluor 633 were recorded at an excitation wavelength of 633 nm and an emission wavelength range of 640–690 nm. The blue images of Hoechst 33342 were recorded at an excitation wavelength of 405 nm and an emission wavelength range of 420–450 nm. Untreated cells were used as the control group.

MCF-7 cells cultured in 4-well glass-bottom dishes were treated with **TDI-COF** (400 μL, 0 or 0.2 mg/mL) for 4 h and exposed to X-ray radiation (0, 2, or 4 Gy). After an additional 30 min of incubation, the cells were fixed in paraformaldehyde (400 μL, 4 wt%) for 24 h at 4°C and washed three times with PBS. The cells were permeabilized with Triton X-100 (400 μL, 0.5 vol%) for 5 min and washed twice with PBS. The cells were incubated in PBS (400 μL) containing normal goat serum (10 vol%) and Tween-20 (0.1 vol%) for 1 h at room temperature. The cells were incubated with rabbit anti-γH2AX (Ser139) primary antibody (100 μL, 1:400 dilution, Cell Signaling Technology, Cat# 9718) at 4°C for 24 h and washed three times with PBS. The cells were incubated with Alexa Fluor 488-conjugated goat anti-rabbit IgG (H+L) cross-adsorbed secondary antibody (200 μL, 1:500 dilution, Thermo Fisher, Cat# A-11008) for 1 h at room temperature. Finally, the cell nuclei were counterstained with Nuclear Red LCS1 (200 μL, 1.0 μM) for 30 min and washed twice with PBS. Laser scanning confocal fluorescence images were captured. The red images of Nuclear Red LCS1 were recorded at an excitation wavelength of 633 nm and an emission wavelength range of 650–700 nm. The green images of Alexa Fluor 488 were recorded at an excitation wavelength of 488 nm and an emission wavelength range of 500–550 nm. Untreated cells were used as the control group.

Intracellular ROS detection

HCT-116 cells cultured in 4-well glass-bottom dishes were treated with **TDI-COF** (400 μL, 0 or 0.15 mg/mL) for 4 h and exposed to X-ray radiation (0, 4, or 8 Gy). MCF-7 cells cultured in 4-well glass-bottom dishes were treated with **TDI-COF** (400 μL, 0 or 0.1 mg/mL) for 4 h and exposed to X-ray radiation (0, 2, or 4 Gy). After an additional 48 h of incubation, the HCT-116 and MCF-7 cells were loaded with DCFH-DA (200 μL, 20 μM) for 30 min in a CO_2 incubator and then washed twice with PBS. Laser scanning confocal fluorescence images were captured with the green signal recorded at an excitation wavelength of 488 nm and an emission wavelength range of 500–540 nm. Untreated cells were used as the control group.

Lipid peroxidation detection

HCT-116 cells cultured in 6-well plates were treated with **TDI-COF** (2.0 mL, 0 or 0.15 mg/mL) for 4 h and exposed to X-ray radiation (0, 4, or 8 Gy). MCF-7 cells cultured in 6-well plates were treated with **TDI-COF** (2.0 mL, 0 or 0.1 mg/mL) for 4 h and exposed to X-ray radiation (0, 2, or 4 Gy). After an additional 48 h of incubation, the HCT-116 and MCF-7 cells were loaded with Liperfluo (1.0 mL, 2.0 μM) for 1 h in a CO_2 incubator and then washed twice with PBS. The cells were harvested using a trypsin/EDTA solution, resuspended in HBSS, strained through a 70 μm cell strainer, and analyzed using a flow cytometer equipped with a 488 nm laser for excitation and an FITC channel for collection. Untreated cells were used as the negative control group. Cells treated with FIN56 (2.0 mL, 2.0 μM) for 24 h were used as the positive control groups.

Western blotting

HCT-116 cells cultured in 6-well plates were treated with **TDI-COF** (2.0 mL, 0 or 0.15 mg/mL) for 4 h and exposed to X-ray radiation (0 or 4 Gy). After 48 h of additional culture, the adherent cells were washed once with PBS and lysed in RIPA lysis buffer (APExBIO, Cat# K1120) supplemented with protease inhibitor cocktail (MedChemExpress, Cat# HY-K0010) and phosphatase inhibitor cocktail (MedChemExpress, Cat# HY-K0022) at 4°C. The lysates were centrifuged at 13000 rpm at 4°C for 15 min, and the supernatant was isolated to exclude debris. The protein concentration was quantified using a BCA protein assay kit (Thermo Fisher, Cat# 23227). The samples were prepared with SDS-PAGE sample loading buffer (Beyotime, Cat# P0015) at 95°C for 15 min, and equal amounts

of protein per sample were loaded onto PAGE gels (Epizyme, Cat# PG112) for electrophoresis to separate the target protein. After transferring the protein to a polyvinylidene difluoride membrane and blocking for 1 h using nonfat powdered milk (5 wt%) in TBST, the membranes were incubated with primary antibodies at 4°C overnight. The primary antibodies used here were rabbit anti-SLC7A11 (1:1000 dilution, Cell Signaling Technology, Cat# 12691), rabbit anti-ACSL4 (1:10000 dilution, Abcam, Cat# ab155282), rabbit anti-GPX4 (1:1000 dilution, Abcam, Cat# ab125066), and rabbit anti-β-tubulin (1:1000 dilution, HuaBio, Cat# ET1602-4). Subsequently, the membranes were washed three times for 10 min in TBST and incubated with HRP-conjugated goat anti-rabbit IgG (H+L) secondary antibody (1:10000 dilution, Proteintech, Cat# SA00001-2) for 1 h at 25°C. The protein of interest was observed using a chemiluminescence detection kit (Vazyme, Cat# E412-01).

Intracellular GSH and MDA measurements

HCT-116 cells cultured in T175 flasks were treated with **TDI-COF** (10 mL, 0 or 0.15 mg/mL) for 4 h and exposed to X-ray radiation (0, 4, or 8 Gy). MCF-7 cells cultured in T175 flasks were treated with **TDI-COF** (10 mL, 0 or 0.1 mg/mL) for 4 h and exposed to X-ray radiation (0 or 4 Gy). After an additional 48 h of incubation, the HCT-116 and MCF-7 cells were carefully rinsed with PBS and used for GSH and MDA measurements according to the manufacturer's guidelines. The GSH and MDA contents were normalized to the total protein amount quantified using a BCA protein assay kit. Untreated cells were used as the control group.

Caspase 3 activation assays

HCT-116 cells cultured in 4-well glass-bottom dishes were treated with **TDI-COF** (400 μL, 0 or 0.15 mg/mL) for 4 h and exposed to X-ray radiation (0, 4, or 8 Gy). After an additional 48 h of incubation, the cells were incubated with CellEvent Caspase-3/7 Green (1×) for 1 h according to the manufacturer's guidelines and then loaded with Nuclear Red LCS1 (200 μL, 1.0 μM) for 30 min in a CO_2 incubator. Laser scanning confocal fluorescence images were captured. The green signals were recorded at an excitation wavelength of 488 nm and an emission wavelength range of 500–540 nm. The red images of Nuclear Red LCS1 were excited by 633 nm light, and the emission wavelength range was collected at 640–690 nm. Untreated cells were used as the negative control group. Cells treated with staurosporine (400 μL, 2.0 μM) for 24 h were used as the positive control groups.

Cell death rescue experiments

HCT-116 cells cultured in 96-well plates were treated with **TDI-COF** (100 μL, 0 or 0.15 mg/mL) for 4 h and exposed to X-ray radiation (0, 4, or 8 Gy). The cells were incubated in a CO_2 incubator for 48 h in the presence of DMSO (100 μL, 0.1 vol%), GSH-EE (100 μL, 1.0 mM), NAC (100 μL, 5.0 mM), DFO (100 μL, 100 μM), Fer-1 (100 μL, 1.0 μM), zVAD (100 μL, 50 μM), Nec-1S (100 μL, 0.5 μM), 3-MA (100 μL, 50 μM). Subsequently, the cells were washed three times with PBS. Then, RPMI-1640 (90 μL) and CCK-8 solution (10 μL) were added to each well, and the plate was incubated in a CO_2 incubator for approximately 1 h. The absorbance at 450 nm was measured using a multimode microplate detection system. Untreated cells were used as the control group.

MCF-7 cells cultured in 96-well plates were treated with **TDI-COF** (100 μL, 0 or 0.1 mg/mL) for 4 h and exposed to X-ray radiation (0 or 4 Gy). The cells were incubated in a CO_2 incubator for 48 h in the presence of DMSO (100 μL, 0.1 vol%), GSH-EE (100 μL, 2.0 mM), NAC (100 μL, 5.0 mM), DFO (100 μL, 100 μM), Fer-1 (100 μL, 1.0 μM), zVAD (100 μL, 50 μM), Nec-1S (100 μL, 0.5 μM), 3-MA (100 μL, 50 μM). Subsequently, the cells were washed three times with PBS. Then, DMEM (90 μL) and CCK-8 solution (10 μL) were added to each well, and the plate was incubated in a CO_2 incubator for approximately 1 h. The absorbance at 450 nm was measured using a multimode microplate detection system. Untreated cells were used as the control group.

Xenograft model

BALB/c nude mice (aged 4 weeks, female) were purchased from Beijing Vital River Laboratory Animal Technology Co., Ltd. The nude mice were housed in a filter-topped facility (pathogen-free) on autoclaved food and water and kept in a room on a 12:12 h light-dark cycle with a temperature between 20–23°C and 30–70% relative humidity.

All animal experiments complied with the relevant guidelines of the Chinese government and regulations for the care and use of

experimental animals under approval number AEECSDNU2021009 by the Ethics Committee of Shandong Normal University (Jinan, China).

HCT-116 cells (approximately 5×10^6 cells) suspended in HBSS (50 μL) were subcutaneously injected into the flanks of each nude mouse to establish the HCT-116 xenograft model. The length (L) and width (W) of the tumor were determined using digital calipers. The tumor volume (V) was calculated using the formula $V = LW^2/2$. Nude mice that failed to develop tumors from the beginning of the experiment were excluded.

Tumor retention

When the tumor reached 6–7 mm in diameter, the mice were randomly divided into 3 groups. Group 1 was intratumorally injected with **TDI-COF** (50 μL; 0.5 mg/mL; 1.45 mM, I equiv.). Group 2 was intratumorally injected with iopromide (50 μL; 0.38 mg/mL; 1.45 mM, I equiv.). Group 3 was intratumorally injected with UiO-66(Hf) (50 μL, 0.57 mg/mL; 1.45 mM, Hf equiv.). Some mice were sacrificed at 2, 4, 24, and 72 h after the injection, and the tumor tissues were collected and digested in aqua regia. The iodine and hafnium contents were detected by ICP–MS. Untreated nude mice were used as the blank for iodine measurements.

Urine excreted from Groups 1–3 during the experiment was collected, acidified with aqua regia, and analyzed by ICP-MS using a similar method.

In vivo antitumor experiments

When the tumor size reached approximately 100 mm^3, the mice were randomly divided into 4 groups. Group 1, as a control group, was intratumorally injected with HBSS (50 μL) at day 0. Group 2 was intratumorally injected with **TDI-COF** (50 μL, 0.5 mg/mL) at day 0. Group 3 was intratumorally injected with HBSS (50 μL) at day 0, followed by exposure to X-ray radiation (4 Gy). Group 4 was intratumorally injected with **TDI-COF** (50 μL, 0.5 mg/mL) at day 0, followed by exposure to X-ray radiation (4 Gy). When the tumor size reached 15 mm in either dimension, the *in vivo* antitumor experiment was terminated, and all mice were euthanized. The tumor tissues and major organs were collected and fixed in paraformaldehyde (4 wt%). The fixed tissues were dehydrated and embedded in paraffin following routine methods. The paraffin-embedded tissues were cut in to slices with a thickness of 3–4 μm, deparaffinized, and rehydrated. H&E staining of the slices was performed using an H&E stain kit according to the manufacturer's guidelines. Furthermore, Ki67 immunohistochemical staining of the slices was performed using a polymer-enhanced two-step immunohistochemistry detection system (ZSGB-BIO, Cat# PV-9001) and a diaminobenzidine kit (ZSGB-BIO, Cat# ZLI-9017) following the manufacturer's protocols. The antibodies used in immunohistochemical analysis is rabbit anti-Ki67 (1:1200 dilution, Servicebio, Cat# GB111499).
